# Spatiotemporal Population Growth Patterns and Interactions Among Sympatric Central European Mesocarnivores

**DOI:** 10.3390/life16020261

**Published:** 2026-02-03

**Authors:** Hanna Bijl, Gergely Schally, Miklós Heltai, Mihály Márton, Szilvia Bőti, Sándor Csányi

**Affiliations:** Department of Wildlife Biology and Management, Institute for Wildlife Management and Nature Conservation, Hungarian University of Agriculture and Life Sciences, Páter Károly Str. 1, H-2100 Gödöllő, Hungary; heltai.miklos.gabor@uni-mate.hu (M.H.); marton.mihaly@uni-mate.hu (M.M.); boti.szilvia@uni-mate.hu (S.B.); s.csanyi@gmail.com (S.C.)

**Keywords:** interspecific competition, coexistence, co-occurrence, interference interactions, mesopredators

## Abstract

Understanding interactions among sympatric mesocarnivore populations is essential for making sound management decisions. The golden jackal has rapidly expanded in Europe, raising questions about its potential intraguild effects. Using long-term hunting bag data (1997–2024) from Hungary, we investigated spatiotemporal population trends of the European badger, red fox, and golden jackal. We examined pairwise associations in their annual growth rates. Generalised additive models and Pearson correlation analyses revealed strong species-specific temporal and spatial trends and weak to moderate positive relationships among the species’ population growth rates at the national scale and within regions of high jackal population density. We found no evidence of jackal suppression of foxes or badgers. Additionally, badgers showed the strongest positive association with fox populations. Our large-scale analyses suggest that these mesocarnivores coexist without substantial competitive interference, likely due to local spatial heterogeneity and fine-scale temporal partitioning that are not detectable in annual, broad-scale (national) data. These findings highlight the importance of integrating broad-scale population data with finer-scale behavioural studies to better understand coexistence mechanisms in expanding mesocarnivore assemblages.

## 1. Introduction

Species coexistence has been an important topic in community ecology, as these studies are essential for understanding the diversity and structure of ecological communities [1]. According to the competitive exclusion principle, two ecologically similar species cannot coexist [2]. Consequently, sympatric species can develop different strategies for resource use, resulting in niche differentiation and divergence to reduce interspecific competition [3,4]. This partitioning of resources can be along the temporal, trophic, and spatial dimensions [5,6]. Because of this niche segregation, understanding the patterns of coexistence (or exclusion) and the ecological traits behind these interactions is fundamental for the effective management of ecological communities [7]. Especially the influence of interspecific interactions within the carnivore guild is extensive [8,9] and has been well-studied on local and global scales [7]. Moreover, interspecific interference among mammalian carnivores could affect population growth and viability by reducing the carrying capacity, lowering foraging efficiency, causing direct mortality from killing, and through inverse density-dependency [10], thereby impacting overall intraguild population dynamics.

The European badger (*Meles meles* Linnaeus, 1758), red fox (*Vulpes vulpes* Linnaeus, 1758), and golden jackal (*Canis aureus moreoticus* Geoffroy, 1835) are among the most widespread mesocarnivores in the Northern Hemisphere, frequently occurring in sympatry and overlapping in resource use, creating potential for interspecific interactions. The golden jackal, in particular, has shown a significant expansion in Europe since the 1970s [11], and an exponential increase in Hungary (our study area) since the mid-1990s [12]. Therefore, it is expected that the arrival of the jackal has likely made a profound impact on the mesocarnivore assemblage both nationally and across Europe, for example, by competitive suppression, spatial exclusion, or trophic shifts [13,14].

Despite the ecological importance of interspecific interactions among mesocarnivores, research on the spatiotemporal population dynamics and coexistence patterns of these three species remains limited. Existing studies are often site-specific [15,16,17], focus only on a single pair of species [18,19], or rely primarily on presence-only camera-trapping data to infer co-occurrence patterns [20]. While camera-based approaches offer fine-scale spatial and behavioural insights, they are typically constrained in temporal extent, spatial coverage, and their ability to infer population-level trends or relative abundance. Consequently, long-term, abundance-based analyses at the national scale remain scarce.

Here, we investigated the spatiotemporal population dynamics and pairwise relationships between the European badger, red fox, and golden jackal in Hungary using hunting bag data from 1997 to 2024. Hunting bags provide an index of abundance and are consistent and spatially extensive, providing information over a much broader scale. This allows for interference with population trends over multiple decades, which other methods, such as presence-only camera-trapping data, cannot capture.

We hypothesised a strong negative correlation between jackal and fox growth rates due to interference competition, as they are considered competitors [13]. By contrast, we expected a weaker positive correlation between badgers and foxes, as they are known to coexist and even cohabit setts [21], likely reflecting commensalism or shared responses to environmental drivers. Lastly, there is little information on jackal–badger interactions, aside from observations of non-antagonistic behaviour at the same baiting site [22]. Therefore, we assumed no significant relationship between the jackal and the badger.

## 2. Materials and Methods

### 2.1. Data Collection

Official hunting bag data from 1997 to 2024 were used as occurrence (abundance) records, obtained from Game Management Units in Hungary, and reported annually to the National Game Management Database (NGMD) [23] (Table S1). These data were intersected with the UTM grid system at a 10 × 10 km resolution [24] to obtain a uniform grid covering the country. The European badger was legally protected from 1974 until 2002 [25]; therefore, we used data from 2002 to 2024 for the badger. Hunting bags can be subjected to differences in hunting/sampling effort [26,27]; however, because the reported data are mandatorily and systematically collected in Hungary within a national game management framework, this greatly reduces potential sources of bias [28,29]. Moreover, hunting bags are adjusted in response to observed population trends and subsequent management objectives rather than being set independently of them, and have long been used as a relative index of population change [28].

Since NGMD data collection began, hunting practices, reporting obligations, and management objectives in Hungary have remained structurally consistent, reducing the likelihood that long-term trends in hunting bag sizes reflect cultural or economic shifts. While hunter affinities or species-specific management priorities (e.g., changes in predator control focus or legal status) may influence harvest intensity at local or short-term scales, such effects are unlikely to generate the consistent, long-term spatiotemporal patterns observed here or to systematically bias interspecific relationships across thousands of observations in grid cells over the years. Importantly, previous national-scale studies have successfully used the same hunting bag database to detect changes in population size [12,30]. Lastly, in a separate study using the same database, we found little difference in overall results when using hunting bag data alone and when accounting for sampling effort by including the number of hunters [31].

### 2.2. Data Analysis

To estimate the population changes of the European badger, red fox, and golden jackal, we first calculated the annual finite rate of increase (λ) [32] per grid cell separately for each species, where

(1) $$\lambda =\frac{N_{t+1}}{N_{t}}.$$

Then, we calculated the intrinsic (exponential) rate of increase (*r*) [32] per grid cell and species by taking the natural logarithm of λ:

(2) $$r=\;\log_{e}\lambda .$$

Instances resulting in non-finite *r*-values due to hunting bags being zero were omitted from the analysis.

Moreover, to evaluate the overall trend of population change, we also estimated the regression slope of hunting bag values for each species in each grid cell. Unlike annual growth rates, which capture year-to-year changes, regression slopes summarise longer-term trends and are less sensitive to short-term fluctuations and zero values. This provides an additional, complementary measure of population dynamics.

Analyses were conducted over the long term (entire period) and the short term (last five years), at both the national and regional scales. The short-term period was selected to represent a relatively stable phase in the growth process, during which both the European badger and golden jackal had completed their major expansion phases and were subject to broadly comparable management and reporting conditions across most regions. This allows us to examine recent interspecific dynamics under conditions of widespread species presence/abundance, reducing the influence of early colonisation effects that were in the initial years of the study period. At the regional scale, we selected grid cells covering Somogy and Baranya Counties, which are known for high-density jackal populations (i.e., hotspots) [12]. The calculated *r*-values were averaged for each grid cell over the whole study period and the most recent five years, and these averages were mapped, along with the slopes, to visualise spatial patterns in population growth. We also performed Pearson correlation analyses of the *r*-values and slopes for each species pair, both long- and short-term, nationally and regionally, to assess interactions between species.

Additionally, we fitted two different types of models: (1) one population trend model to understand the spatiotemporal population dynamics of each species, and (2) a set of species-interaction models (one for each species) to test for associations between the annual growth rates of the species.

For the population trend model, we modelled spatial and temporal variation in hunting bags using a generalised additive model (GAM) with a Tweedie error distribution *Tw* (power parameter *p* = 1.453) and log link. The Tweedie power parameter was estimated directly from the data during model fitting using fast restricted maximum likelihood (fREML). The response variable was the annual hunting bag, $y_{gti}$ for species *i* in grid cell *g* during year *t*. The model included species as a categorical predictor $\alpha_{i}$ and allowed both temporal and spatial trends to differ between species by fitting species-specific smooth functions *s* of year and space. Specifically, we included a smooth function of year for each species and a bivariate smooth function of geographic coordinates (longitude and latitude of the grid centroids) for each species:

(3) $$y_{git}\;\sim \;Tw\left(\mu_{git},p,\;\sigma^{2}\right),\;\;{log(\mu }_{git})=\phi_{git}\;\;\phi_{git}=\;\alpha_{i}+s_{i}\left({year}_{t}\right)+s_{i}({lon}_{g},{lat}_{g}).$$

For the species-interaction model, we fitted a separate GAM with Gaussian errors and an identity link for each focal species. For a given focal species, the model included the previously calculated annual growth rates *r* of the other two species in the same grid cell and year as linear fixed effects. To account for residual spatiotemporal structure, we included smooth functions of year and a bivariate smooth of spatial coordinates, similar to the population trend model:

(4) $$r_{jackal,\;gt}=\beta_{0}+\;\beta_{1}r_{badger,gt}+{\beta_{2}r}_{fox,gt}+s\left({year}_{t}\right)+s\left({lon}_{g},{lat}_{g}\right)+\epsilon_{gt},$$

(5) $$r_{fox,\;gt}=\beta_{0}^{\prime }+\beta_{3}r_{badger,gt}+{\beta_{4}r}_{jackal,gt}+s\left({year}_{t}\right)+s\left({lon}_{g},{lat}_{g}\right)+\epsilon_{gt}^{\prime },$$

(6) $$r_{badger,\;gt}=\beta_{0}^{\prime \prime }+\beta_{5}r_{fox,gt}+\beta_{6}r_{jackal,gt}+s\left({year}_{t}\right)+s\left({lon}_{g},{lat}_{g}\right)+\epsilon_{gt}^{\prime \prime }.$$

We ran this model suite twice: first using data from the entire country, and second using data restricted to the two counties with the highest golden jackal density.

The analyses and modelling were performed in R version 4.4.1 [33] using the “mgcv” version 1.9-1 package [34].

## 3. Results

**Figure 1:**
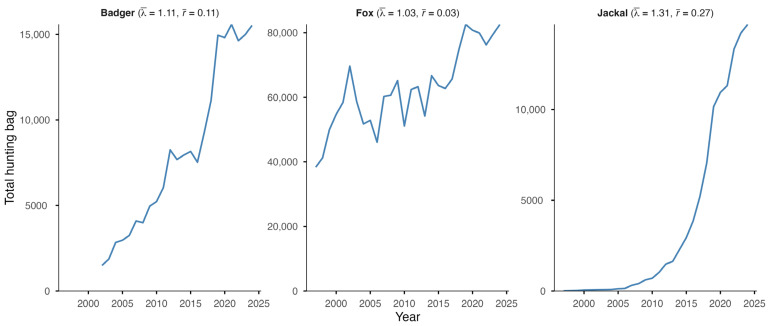
Total hunting bag of the European badger, red fox, and golden jackal in Hungary during 1997–2024 (2002–2024 for the badger) with geometric mean of the annual finite rate of increase ($\bar{\lambda }$) and mean annual intrinsic rate of increase ($\bar{r}$).

**Figure 2:**
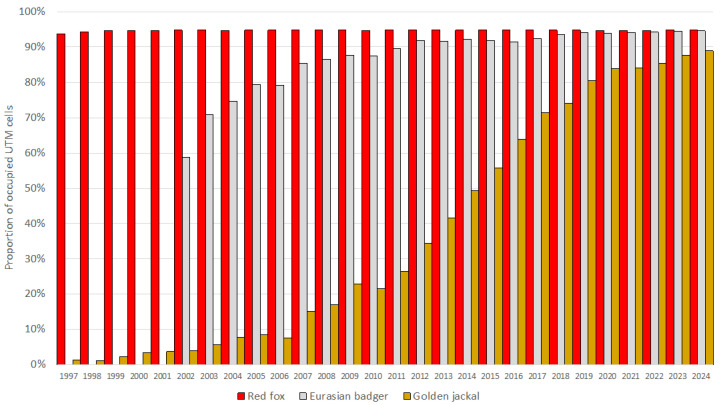
Proportion of occupied UTM grid cells based on hunting bag data of the European badger, red fox, and golden jackal in Hungary during 1997–2024 (2002–2024 for the badger).

**Figure 3:**
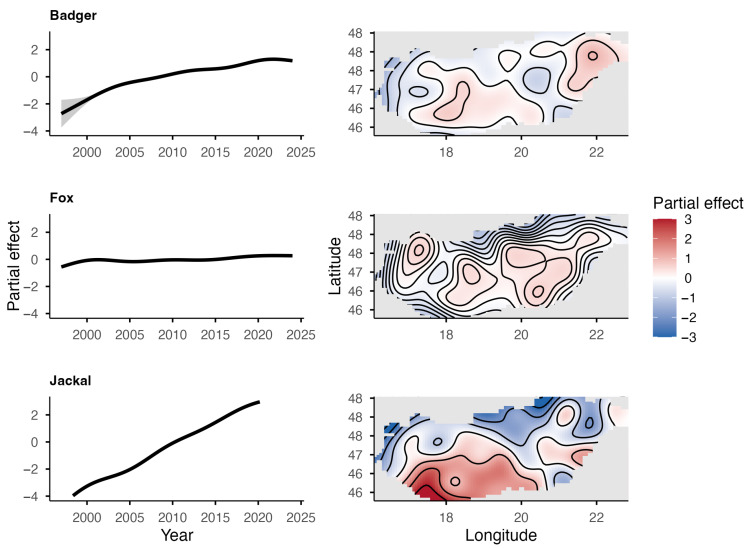
Partial effects of time (**top**) and space (**bottom**) from the GAM for the European badger, red fox, and golden jackal in Hungary based on hunting bag data.

The European badger, red fox, and golden jackal all showed an increase in total hunting bags in Hungary over the study period (Figure 1). The jackal showed the most substantial increase ($\bar{\lambda }$ = 1.31; $\bar{r}$ = 0.27) among the species, followed by the badger ($\bar{\lambda }$ = 1.11; $\bar{r}$ = 0.11), while the fox showed a moderate increase ($\bar{\lambda }$ = 1.03; $\bar{r}$ = 0.03). However, range expansion, through the UTM cell coverage, could only be observed in the badger (59% in 2002 to 95% in 2024) and the golden jackal (1% in 1997 to 89% in 2024) (Figure 2). The range of the red fox had the greatest coverage and remained stable during the studied period. The population trend model showed substantial species-level differences in hunting bag sizes and nonlinear spatiotemporal patterns (Figure 3). Relative to the jackal, both badger and fox exhibited higher hunting bags (*β* = 2.73 and *β* = 5.37, respectively; both *p* < 0.001). Species-specific temporal smooths for the year were highly significant (edf = 6.19 (badger), 6.96 (fox), 6.75 (jackal); all *p* < 0.001), indicating distinct nonlinear trends across the study period. Spatial smooths for longitude and latitude were also significant for all species (edf = 47.69 (badger), 48.54 (fox), 48.51 (jackal); all *p* < 0.001), reflecting firm spatial heterogeneity in hunting intensity with limited spatial overlap among the species. Specifically, the spatial smooth for the badger showed relatively weak and patchy spatial structuring, with alternating areas of slightly positive and negative effects across the country, indicating that the population growth is moderately spatially structured but largely diffuse at the national scale. In contrast, the fox showed a stronger and more spatially coherent pattern, with better-defined regions of positive and negative effects, indicating that the fox population growth was more regional. The spatial smooth for the jackal showed the most significant spatial heterogeneity, with higher predicted hunting bags in the southwest of the country. The model explained 83.2% of the deviance (adj. R^2^ = 0.71), showing that differences among the species, together with spatial and temporal variation, account for most of the observed patterns in hunting bag data.

**Figure 4:**
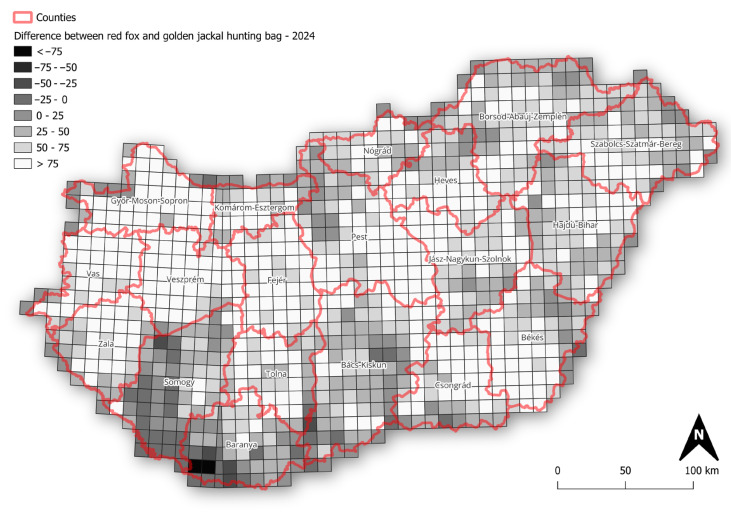
Difference in hunting bag size between red fox and golden jackal across Hungary in 2024 (values represent red fox hunting bag minus golden jackal hunting bag).

**Figure 5:**
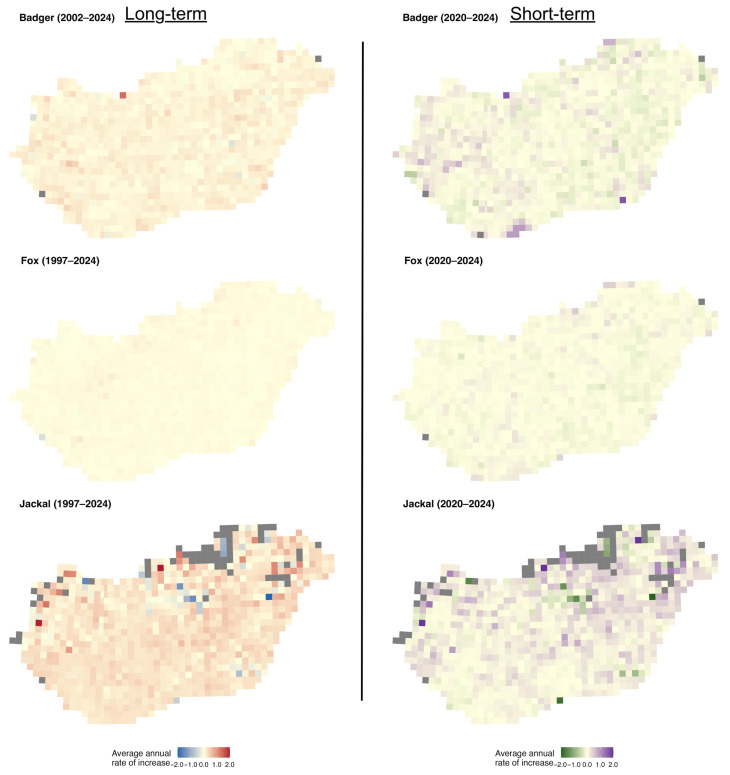
Distribution of average annual rates of increase of the European badger, red fox, and golden jackal in Hungary over the long-term (1997–2024, badger 2002–2024) and short-term periods (2020–2024). Grey grid cells indicate no data.

The mapped *r*-values and slopes indicate that the golden jackal showed the strongest growth among the species, particularly in southwestern Hungary, coinciding with areas where its hunting bag now exceeds that of the red fox (Figure 4), whereas fox and badger trends were more geographically uniform. Specifically, over the long term, badger growth rates were spatially diffuse and rather weakly structured, with most grids showing modest positive or near-stable growth (Figure 5). In the short term, mean *r*-values become more spatially heterogeneous, with small localised patches of positive and negative growth, suggesting a minor increase in spatial variability in recent years. Mean growth rates for the fox showed strong uniformity over the long term, with the majority of grid cells near neutral or weakly positive. Short-term mean *r*-values remain similarly homogeneous, indicating broad population stability at the national scale with limited spatial differentiation. In the case of the jackal, the long-term *r*-values showed clear spatial structuring, with particularly high values in the north and near-neutral values elsewhere in the country. In the short term, the mean *r*-values become more neutral, with stronger values in the northern and eastern parts of the country.

**Figure 6:**
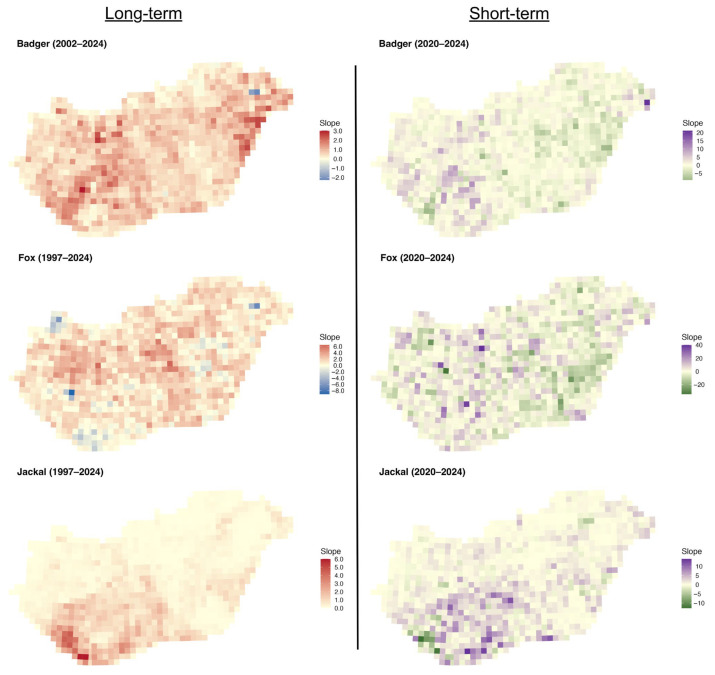
Distribution of regression slopes of hunting bags on year for the European badger, red fox, and golden jackal in Hungary over the long-term (1997–2024, badger 2002–2024) and short-term periods (2020–2024).

The long-term slopes for the badger were mainly positive across the country, with stronger trends in the eastern and central-western parts (Figure 6). The short-term slopes were more mixed, with small areas of decline interspersed with positive trends in the western part. For the fox, the long-term slopes were mainly positive across the country, with small patches of declines. Short-term slopes, however, display greater spatial noise, with small patches of positive and negative values. For the golden jackal, both the long-term and short-term slopes showed a substantial increase in the southwestern part of the country, with tiny patches of decline in the short term.

**Figure 7:**
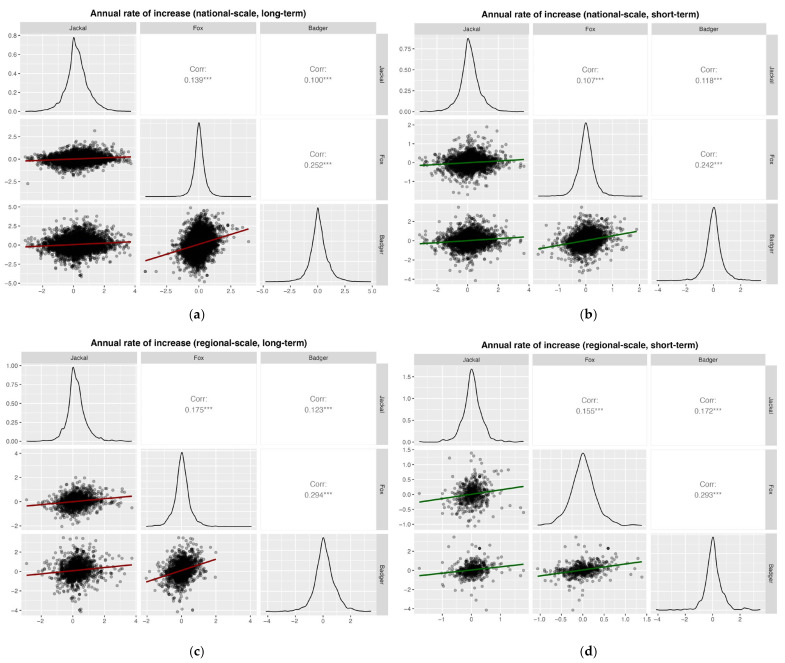
Pearson correlation analyses based on the annual rates of increase for (**a**) national, long-term (1997–2024; badger 2002–2024), (**b**) national, short-term (2020–2024); (**c**) regional, long-term (1997–2024, badger 2002–2024); and (**d**) regional, short-term (2020–2024). Significance levels: * *p* < 0.05; ** *p* < 0.01; *** *p* < 0.001.

Pearson correlation analyses based on annual rates of increase revealed weakly significant positive correlations among all species pairs at national and regional scales, for both long- and short-term periods (all Pearson’s *r* < 0.3; Figure 7).

**Figure 8:**
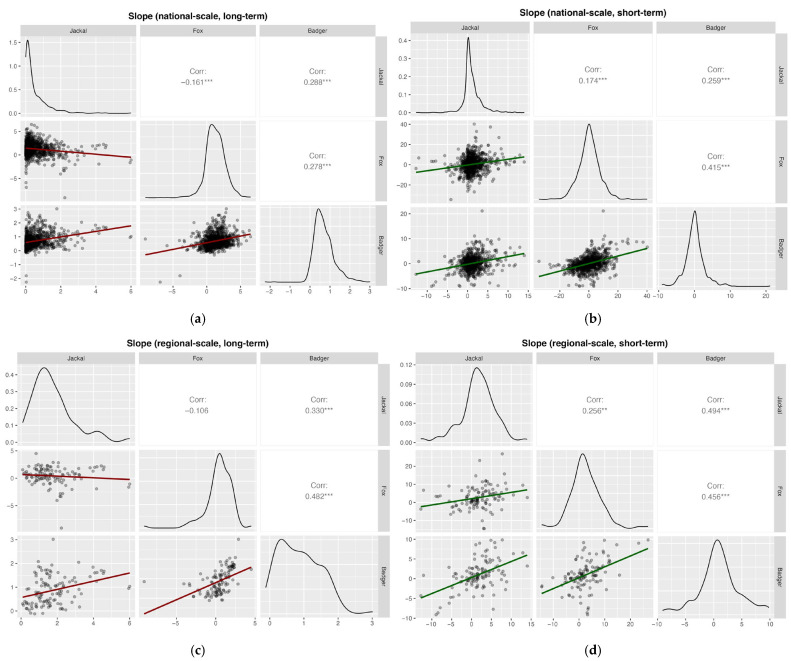
Pearson correlation analyses based on slopes of hunting bags on year for (**a**) national, long-term (1997–2024, badger 2002–2024) (**b**) national, short-term (2020–2024); (**c**) regional, long-term (1997–2024, badger 2002–2024); and (**d**) regional, short-term (2020–2024). Significance levels: * *p* < 0.05; ** *p* < 0.01; *** *p* < 0.001.

Pearson correlation analyses based on the slopes showed weak but significant positive correlations for most species’ pairs across spatial scales and time periods (Pearson’s *r* < 0.3), except for jackal–fox in the long-term at both national (Pearson’s *r* = −0.16) and regional scales (Pearson’s *r* = −0.11; Figure 8). Furthermore, fox–badger correlations were generally moderate and positive across all analyses, except in the long-term national case.

Lastly, across all three species-interaction models fitted to data from the entire country, the parametric terms revealed strong positive associations among the annual rates of increase of badger, fox, and jackal (Table A1). When modelling badger growth rates, both fox and jackal were significant positive predictors (*β* = 0.51 and *β* = 0.06, respectively; both *p* < 0.001). Similarly, fox growth rates were positively associated with badger and jackal growth (*β* = 0.12 and *β* = 0.04, respectively; both *p* < 0.001). Finally, in models of jackal growth, both badger and fox were significant positive predictors (*β* = 0.07 and *β* = 0.23, respectively; both *p* < 0.001). Especially the badger showed the strongest (positive) association with the fox.

Despite these consistent cross-species associations, the smooth terms showed only modest nonlinear temporal and spatial effects, with year explaining limited additional variation (edf = 9.77 (badger), 13.79 (fox), 6.43 (jackal); all *p* < 0.001) and spatial patterns capturing small but significant spatial heterogeneity (edf = 2.80 (badger), 6.09 (fox), 2.00 (jackal); all *p* < 0.001). Overall, the models explained a relatively low proportion of deviance (jackal model: 3.9%, adj. R^2^ = 0.04; fox model: 11.7%, adj. R^2^ = 0.11; badger model: 9.3%, adj. R^2^ = 0.09). While the associations are statistically significant, they explain very little of the observed variation in growth rates, indicating weak relationships among the species’ annual growth rates and a limited contribution of interspecific interactions to population dynamics.

The species-interaction models fitted for the high golden jackal density region also yielded similar results (Table A1). Badger and fox were significant positive predictors of jackal growth rates (*β* = 0.06 and *β* = 0.17, respectively; both *p* < 0.001), badger and jackal similarly predicted fox growth rates (*β* = 0.14 and *β* = 0.07, respectively; both *p* < 0.001), and fox and jackal predicted badger growth rates (*β* = 0.59; *p* < 0.001 and *β* = 0.10, *p* < 0.01, respectively). The effect sizes were generally stronger at the regional scale compared to the national-scale model, except for badger on jackal (*β* = 0.07 to 0.06) and fox on jackal (*β* = 0.23 to 0.17) interactions, which became weaker. Notably, there was no evidence of jackal suppression of foxes or badgers in high-density regions. In fact, badgers appeared to respond more strongly to foxes than to jackals.

The smooth terms indicated modest temporal nonlinearity (edf = 17.58 (badger), 16.90 (fox), 18.73 (jackal); all *p* < 0.001) and generally weak spatial effects (edf = 2.00; *p* = 0.125 (badger), 2.00; *p* = 0.914 (fox), 2.00; *p* < 0.01 (jackal)). These models also had a relatively low proportion of deviance (badger model: 12.9%, adj. R^2^ = 0.12; fox model: 16.9%, adj. R^2^ = 0.16; jackal model: 13.6%, adj. R^2^ = 0.13), indicating no competitive interference among the species either at the national or regional scale.

## 4. Discussion

We found that populations of the European badger, red fox, and golden jackal increased over the last three decades. However, despite the arrival of the golden jackal and its expansion in Hungary since the mid-1990s, we found no evidence of jackal suppression of foxes or badgers at either national or regional scales, across long- or short-term periods.

Pearson correlation analyses revealed statistically significant associations between species growth rates and regression slopes across spatial scales and time periods; however, effect sizes were consistently small and remained correlational. Given the large number of observations per grid cell per year, these statistical significances should be interpreted with caution. Moreover, these weak correlations indicate limited ecological coupling among species and likely indicate shared (synchronous) external responses to broad-scale drivers, rather than strong interspecific dependence. This is consistent with the low explained variation in the growth rates. Hence, including environmental covariates (e.g., land cover, prey availability, climate variables) would improve our understanding of the combined main drivers of population growth for these mesocarnivores. Additionally, the contrast between (weak) positive correlations in annual growth rates and (weak) negative correlations in long-term slopes for jackal-fox interactions likely reflects different temporal processes captured by these metrics, rather than direct competition.

Furthermore, while competitive effects between species might be lagged, meaning that an increase in one species could influence the growth of another in the following year(s), the use of long-term annual growth rates rather than absolute abundances means that contemporaneous associations still provide essential information on interspecific dynamics over time. Accordingly, same-year models provide an appropriate representation of the interspecific interactions at the annual scale, without requiring stronger assumptions about the timing of specific temporal delays between interspecific interactions and their demographic consequences.

One explanation for the observed positive associations among mesocarnivores may be synchronous management (predator control) within the country. Historically, Hungarian legislation permitted extensive predator removal (Act VI of 1872 and Act XX of 1883), including the award of bounties (rewarding the confirmed culls). In contrast, predator control was loosened mainly during the second half of the 20th century due to increased legal protections, restrictions on hunting methods, and the spread of small-game breeding [35]. Currently, management is focused on reducing predator populations to minimise potential human-carnivore conflict and protect declining small game populations [36,37], as well as reducing predation on roe deer (*Capreolus capreolus* Linnaeus, 1758) fawns [38].

Alternatively, the positive associations in growth rates may reflect spatial heterogeneity, where inferior competitors select landscape features where dominant species are less common or where cover provides protection [39]; patterns that are not detectable at broader spatial scales, as in our study. This interpretation aligns with evidence of local avoidance and regional coexistence, driven by environmental spatial structuring [1]. Coexistence may also arise when predator-prey ecological parameters (i.e., characteristics that determine how species interact quantitatively) fall within tolerable ranges and coexistence is facilitated by periodic behaviours [40], such as diel activity shifts, rather than just spatial segregation. These processes are particularly plausible when key prey species, such as rodents, provide an unlimited food supply, thereby reducing direct competition for (abundant) resources. Overall, spatial heterogeneity and temporal partitioning as mechanisms of coexistence support the ‘niche variation hypothesis’, in which populations of sympatric species become more generalised when released from competitors [41,42].

Differences in trophic niches, (seasonal) dietary shifts, and species-specific behaviours may also support coexistence among these mesocarnivores, especially since all three species are considered generalists, which facilitates trophic niche partitioning. In particular, the golden jackal and red fox feed predominantly on small mammals, but the fox consumes higher proportions of birds, indicating partial dietary separation between the two species [19,43]. The highest overlap was found when the species preyed upon rodents [44]. Overall, foxes are more generalists (broader trophic niche) than jackals, and hunt mainly in solitude [13,19]. Similarly, European badgers consume a wide range of prey/food items [45], including earthworms, insects, birds, small mammals, domestic plants, and anthropogenic food sources [46,47]. Given these high trophic niche overlaps, spatiotemporal partitioning may be key to fox–jackal sympatry [44]. Especially among mesocarnivores with high spatial and temporal overlap, fine-scale temporal partitioning was found to be the primary mechanism facilitating coexistence [6]. However, context-specific factors (e.g., carcass possession, individual experience, body condition) may change established dominance hierarchies [48]. For example, in Greece, jackals have been observed submitting to foxes under competitive conditions, which is the first documented case of submissive behaviour [48]. In contrast, in Israel, red fox populations decreased in areas with high jackal abundance [14], and showed avoidance in core activity areas of jackals and restricted their activity to their peripheries [49].

Badgers, on the other hand, did not show agonistic behaviour towards jackals [22] or towards Iberian lynx (*Lynx pardinus* Temminck, 1827), suggesting possible commensalism [50], whereas Eurasian lynx (*Lynx lynx* Linnaeus, 1758) can suppress red foxes [51]. Moreover, our results support the existing positive relationship between foxes and badgers; they have similar temporal activity patterns and show no spatiotemporal avoidance [52]. Through habitat amelioration and refuge from cold and predation, badgers facilitate not only foxes but also raccoon dogs (*Nyctereutes procyonoides* Gray, 1834) [53], which are present in Hungary, albeit in lower numbers than other mesocarnivores [30]. However, there was a lower proportion of active badger sett entrances found where foxes or raccoon dogs were present [54], and the species pair differed in night-time activity in all seasons [55].

Other mesocarnivore guilds in different areas showed similar patterns. For example, in North America, the mesocarnivore guild is shaped mainly by habitat and scale-dependent processes, with direct competitive interactions playing a limited and context-specific role [56]. There was no evidence found for competition between bobcats (*Lynx rufus* Schreber, 1777), coyotes (*Canis latrans* Say, 1823), and red foxes [57], or between coyotes and swift foxes (*Vulpes velox* Say, 1823) [58].

Overall, these interactions suggest that coexistence and competition are context-dependent outcomes along ecological axes, such as resource abundance and population density, ranging from commensalism/facilitation to competition, depending on local conditions and behavioural flexibility [59]. When resources are abundant or predictable, overlap among species is more tolerated, whereas resource limitations or increasing densities can intensify competitive interactions. The weak effect sizes and low explanatory power observed in our models suggest that, at the national scale, mesocarnivore dynamics are mainly shaped by common external drivers or management objectives, with competitive effects playing a secondary, context-specific role.

## 5. Conclusions

We found no evidence of fox or badger suppression by jackals, whereas badgers showed the strongest positive association with foxes. Overall, the spatiotemporal growth patterns and interactions among the European badger, red fox, and golden jackal suggest that local behavioural avoidance or interference competition may occur, but that these fine-scale processes are likely diluted at larger scales, leading to apparent coexistence at regional or national scales. Integrating fine-scale behavioural data (e.g., camera trapping, telemetry) with landscape-level analyses would facilitate a more mechanistic understanding of coexistence and competition among (expanding) mesocarnivores. In particular, integrative frameworks such as integrated population models, which combine hunting bag data with telemetry-derived survival estimates or camera trap occupancy data, could help bridge scales and clarify underlying processes. In addition, multi-species hierarchical models that jointly estimate species responses while sharing spatial and temporal random effects could provide further insight into interspecific dynamics.

Lastly, given that carnivore populations interact in complex ways and assemblage structure is shaped by fine-scale processes [55], our results highlight the need for adaptive management that considers the whole (meso)carnivore guild rather than single species. Importantly, field studies should not only focus on cases of intraguild competition but also on its absence, especially since the coexistence of species within a guild is necessary for biodiversity to exist in the first place [10].

## Data Availability

The data presented in this study are available in the Supplementary Information.

## Supplementary Materials

The following supporting information can be downloaded at: https://www.mdpi.com/article/10.3390/life16020261/s1, Table S1: Hunting bag data for European badger, red fox, and golden jackal in Hungary (1997–2024).

## Abbreviations

The following abbreviations are used in this manuscript:

GAM	Generalised additive model
NGMD	National Game Management Database (also known as Hungarian Game Management Database)

## Appendix A

**Table A1:** Summary of the species-interaction GAM based on annual rates of increase at the national scale (national) and within high jackal-density regions (regional).

Model	Term	Estimate	Std Error	Statistic	*p*-Value	edf	Ref df
National							
*r*_jackal_	(intercept)	0.227	0.008	29.354	<0.001		
	*r*_badger_	0.071	0.012	6.108	<0.001		
	*r*_fox_	0.232	0.024	9.719	<0.001		
	s (year)			20.891	<0.001	6.427	7.451
	s (lon, lat)			9.216	<0.001	2.000	2.000
*r*_fox_	(intercept)	0.005	0.004	1.348	0.178		
	*r*_badger_	0.116	0.005	23.669	<0.001		
	*r*_jackal_	0.043	0.005	9.240	<0.001		
	s (year)			22.240	<0.001	13.794	13.986
	s (lon, lat)			3.819	<0.001	6.092	8.432
*r*_badger_	(intercept)	0.058	0.007	7.88	<0.001		
	*r*_fox_	0.513	0.021	23.92	<0.001		
	*r*_jackal_	0.059	0.010	6.09	<0.001		
	s (year)			11.22	<0.001	9.774	11.388
	s (lon, lat)			7.52	<0.001	2.803	3.451
Regional							
*r*_jackal_	(intercept)	0.208	0.013	15.785	<0.001		
	*r*_badger_	0.056	0.018	3.189	<0.01		
	*r*_fox_	0.174	0.036	4.798	<0.001		
	s (year)			11.163	<0.001	18.73	18.99
	s (lon, lat)			3.517	<0.05	2.00	2.00
*r*_fox_	(intercept)	−0.006	0.009	−0.676	0.499		
	*r*_badger_	0.139	0.011	12.724	<0.001		
	*r*_jackal_	0.072	0.015	4.822	<0.001		
	s (year)			6.746	<0.001	16.90	18.41
	s (lon, lat)			0.090	0.914	2.00	2.00
*r*_badger_	(intercept)	0.079	0.018	4.240	<0.001		
	*r*_fox_	0.589	0.046	12.690	<0.001		
	*r*_jackal_	0.098	0.031	3.175	<0.01		
	s (year)			2.533	<0.001	17.58	18.71
	s (lon, lat)			2.080	0.125	2.00	2.00

## References

[1] Monterroso P., Díaz-Ruiz F., Lukacs P.M., Alves P.C., Ferreras P. (2020). Ecological Traits and the Spatial Structure of Competitive Coexistence among Carnivores. Ecology.

[2] Holt R.D. (2017). Species Coexistence. Reference Module in Life Sciences.

[3] Tilman D. (1987). The Importance of the Mechanisms of Interspecific Competition. Am. Nat..

[4] Friedemann G., Leshem Y., Kerem L., Shacham B., Bar-Massada A., McClain K.M., Bohrer G., Izhaki I. (2016). Multidimensional Differentiation in Foraging Resource Use during Breeding of Two Sympatric Top Predators. Sci. Rep..

[5] Ferreiro-Arias I., Isla J., Jordano P., Benítez-López A. (2021). Fine-Scale Coexistence between Mediterranean Mesocarnivores Is Mediated by Spatial, Temporal, and Trophic Resource Partitioning. Ecol. Evol..

[6] Barros A.L., Raposo D., Almeida J.D., Jesus H., Oliveira M.A., Fernandes C.R., MacKenzie D.I., Santos-Reis M. (2024). An Integrated Assessment of Niche Partitioning Reveals Mechanisms of Coexistence between Mesocarnivores. Glob. Ecol. Conserv..

[7] Davis C.L., Rich L.N., Farris Z.J., Kelly M.J., Di Bitetti M.S., Blanco Y.D., Albanesi S., Farhadinia M.S., Gholikhani N., Hamel S. (2018). Ecological Correlates of the Spatial Co-Occurrence of Sympatric Mammalian Carnivores Worldwide. Ecol. Lett..

[8] Rosenzweig M.L. (1966). Community Structure in Sympatric Carnivora. J. Mammal..

[9] Palomares F., Caro T.M. (1999). Interspecific Killing among Mammalian Carnivores. Am. Nat..

[10] Linnell J.D.C., Strand O. (2000). Interference Interactions, Coexistence and Conservation of Mammalian Carnivores. Divers. Distrib..

[11] Arnold J., Humer A., Heltai M., Murariu D., Spassov N., Hackländer K. (2012). Current Status and Distribution of Golden Jackals *Canis aureus* in Europe. Mammal. Rev..

[12] Bijl H., Schally G., Márton M., Heltai M., Csányi S. (2024). From Invaders to Residents: The Golden Jackal (*Canis aureus*) Expansion in Hungary since the Mid-1990s. PLoS ONE.

[13] Lanszki J., Heltai M., Szabo L. (2006). Feeding Habits and Trophic Niche Overlap between Sympatric Golden Jackal (*Canis aureus*) and Red Fox (*Vulpes vulpes*) in the Pannonian Ecoregion (Hungary). Can. J. Zool..

[14] Scheinin S., Yom-Tov Y., Motro U., Geffen E. (2006). Behavioural Responses of Red Foxes to an Increase in the Presence of Golden Jackals: A Field Experiment. Anim. Behav..

[15] Tsunoda H., Ito K., Peeva S., Raichev E., Kaneko Y. (2018). Spatial and Temporal Separation between the Golden Jackal and Three Sympatric Carnivores in a Human-Modified Landscape in Central Bulgaria. Zool. Ecol..

[16] Tsunoda H., Newman C., Peeva S., Raichev E., Buesching C.D., Kaneko Y. (2020). Spatio-Temporal Partitioning Facilitates Mesocarnivore Sympatry in the Stara Planina Mountains, Bulgaria. Zoology.

[17] Jaklič A., Potočnik H. (2022). Interspecific Interactions between Golden Jackals (*Canis aureus*) and Other Mesocarnivores at Bait Stations in Ljubljansko Barje. Nat. Slov..

[18] Macdonald D.W., Buesching C.D., Stopka P., Henderson J., Ellwood S.A., Baker S.E. (2004). Encounters between Two Sympatric Carnivores: Red Foxes (*Vulpes vulpes*) and European Badgers (*Meles meles*). J. Zool..

[19] Torretta E., Riboldi L., Costa E., Delfoco C., Frignani E., Meriggi A. (2021). Niche Partitioning between Sympatric Wild Canids: The Case of the Golden Jackal (*Canis aureus*) and the Red Fox (*Vulpes vulpes*) in North-Eastern Italy. BMC Ecol. Evol..

[20] Barrull J., Mate I., Ruiz-Olmo J., Casanovas J.G., Gosàlbez J., Salicrú M. (2014). Factors and Mechanisms That Explain Coexistence in a Mediterranean Carnivore Assemblage: An Integrated Study Based on Camera Trapping and Diet. Mamm. Biol..

[21] Nowakowski K., Ważna A., Kurek P., Cichocki J., Gabryś G. (2020). Reproduction Success in European Badgers, Red Foxes and Raccoon Dogs in Relation to Sett Cohabitation. PLoS ONE.

[22] Konstantinov Y., Spassov N., Acosta-Pankov I. (2022). First Records of Golden Jackal and European Badger Non-Antagonistic Interaction at the Lower Danube (Bulgaria). North-West J. Zool..

[23] Csányi S., Lehoczki R., Sonkoly K. (2010). National Game Management Database of Hungary. Int. J. Inf. Syst. Soc. Change (IJISSC).

[24] Horváth F., Molnár Z., Bölöni J., Pataki Z., Polgár L., Révész A., Oláh K., Krasser D., Illyés E. (2008). Fact Sheet of the MÉTA Database 1.2. Acta Bot. Hung..

[25] Márton M., Markolt F., Szabó L., Kozák L., Lanszki J., Patkó L., Heltai M. (2016). Den Site Selection of the European Badger, *Meles meles* and the Red Fox, *Vulpes vulpes* in Hungary. Folia Zool..

[26] Ranta E., Lindström J., Lindén H., Helle P. (2008). How Reliable Are Harvesting Data for Analyses of Spatio-Temporal Population Dynamics?. Oikos.

[27] Willebrand T., Hörnell-Willebrand M., Asmyhr L. (2011). Willow Grouse Bag Size Is More Sensitive to Variation in Hunter Effort than to Variation in Willow Grouse Density. Oikos.

[28] Consortium E., Vicente J., Plhal R., Blanco-Aguiar J.A., Sange M., Podgórski T., Petrovic K., Scandura M., Nabeiro A.C., Body G. (2018). Analysis of Hunting Statistics Collection Frameworks for Wild Boar across Europe and Proposals for Improving the Harmonisation of Data Collection. EFSA Support. Publ..

[29] Ruiz-Rodríguez C., Blanco-Aguiar J.A., Gómez-Molina A., Illanas S., Fernández-López J., Acevedo P., Vicente J. (2023). Towards Standardising the Collection of Game Statistics in Europe: A Case Study. Eur. J. Wildl. Res..

[30] Schally G., Bijl H., Kashyap B., Márton M., Bőti S., Katona K., Biró Z., Heltai M., Csányi S., Schally G. (2024). Analysis of the Raccoon (*Procyon lotor*) and Common Raccoon Dog (*Nyctereutes procyonoides*) Spatiotemporal Changes Based on Hunting Bag Data in Hungary. Diversity.

[31] Bijl H., Schally G., Heltai M., Emami-Khoyi A., Csányi S. (2025). Multi-Phase Modelling of Potential Drivers for the Range Expansion of the Golden Jackal (*Canis aureus*). Sci. Rep..

[32] Caughley G. (1977). Analysis of Vertebrate Populations.

[33] R Core Team (2024). R: A Language and Environment for Statistical Computing.

[34] Wood S.N. (2011). Fast Stable Restricted Maximum Likelihood and Marginal Likelihood Estimation of Semiparametric Generalized Linear Models. J. R. Stat. Soc. Ser. B Stat. Methodol..

[35] Heltai M. (2010). Emlős Ragadozók Magyarországon.

[36] Baines D., Fletcher K., Hesford N., Newborn D., Richardson M. (2023). Lethal Predator Control on UK Moorland Is Associated with High Breeding Success of Curlew, a Globally near-Threatened Wader. Eur. J. Wildl. Res..

[37] Baines D. (2025). Ten Years on from a Predator Removal Experiment in the English Uplands: Changes in Numbers of Ground-Nesting Birds and Predators. J. Nat. Conserv..

[38] Jarnemo A., Liberg O. (2005). Red Fox Removal and Roe Deer Fawn Survival—A 14-Year Study. J. Wildl. Manage.

[39] Davies A.B., Tambling C.J., Marneweck D.G., Ranc N., Druce D.J., Cromsigt J.P.G.M., le Roux E., Asner G.P. (2021). Spatial Heterogeneity Facilitates Carnivore Coexistence. Ecology.

[40] Muratori S., Rinaldi S. (1989). Remarks on Competitive Coexistence. SIAM J. Appl. Math..

[41] Schoener T.W. (1974). Resource Partitioning in Ecological Communities. Science.

[42] Bolnick D.I., Svanbäck R., Araújo M.S., Persson L. (2007). Comparative Support for the Niche Variation Hypothesis That More Generalized Populations Also Are More Heterogeneous. Proc. Natl. Acad. Sci. USA.

[43] Lanszki J., Kurys A., Szabó L., Nagyapáti N., Porter L.B., Heltai M. (2016). Diet Composition of the Golden Jackal and the Sympatric Red Fox in an Agricultural Area (Hungary). Folia Zool..

[44] Tsunoda H. (2022). Niche Overlaps and Partitioning Between Eurasian Golden Jackal *Canis aureus* and Sympatric Red Fox *Vulpes vulpes*. Proc. Zool. Soc..

[45] Roper T.J. (1994). The European Badger *Meles meles*: Food Specialist or Generalist?. J. Zool..

[46] Remonti L., Balestrieri A., Prigioni C. (2011). Percentage of Protein, Lipids, and Carbohydrates in the Diet of Badger (*Meles meles*) Populations across Europe. Ecol. Res..

[47] Gomes D.J., Wierzbowska I.A., Bevanger K., O’Mahony D.T., Rola K. (2019). Diet of the European Badgers (*Meles meles*) in Urban and Rural Areas of Norway. Eur. J. Wildl. Res..

[48] Zevgolis Y.G., Kotselis C., Giritziotis B., Lekka A., Christopoulos A. (2025). Subverting Dominance Hierarchies: Interspecific Submission and Agonistic Interactions Between Golden Jackals and a Red Fox. Diversity.

[49] Shamoon H., Saltz D., Dayan T. (2017). Fine-Scale Temporal and Spatial Population Fluctuations of Medium Sized Carnivores in a Mediterranean Agricultural Matrix. Landsc. Ecol..

[50] Pérez-Vigo I., Ferreras P., Finat R., Villafuerte R. (2025). Tolerance and Intraguild Commensalism: The Case of the European Badger and the Iberian Lynx. Eur. J. Wildl. Res..

[51] Pasanen-Mortensen M., Pyykönen M., Elmhagen B. (2013). Where Lynx Prevail, Foxes Will Fail—Limitation of a Mesopredator in Eurasia. Glob. Ecol. Biogeogr..

[52] Oliveira R., Lazzeri L., Mouton R., Gomez V., Ferretti F. (2025). Temporal Relationships between the Red Fox and the European Badger in a Mediterranean Protected Area. J. Zool..

[53] Kowalczyk R., Jędrzejewska B., Zalewski A., Jędrzejewski W. (2008). Facilitative Interactions between the Eurasian Badger (*Meles meless*), the Red Fox (*Vulpes vulpes*), and the Invasive Raccoon Dog (*Nyctereutes procyonoides*) in Białowieża Primeval Forest, Poland. Can. J. Zool..

[54] Tammeleht E., Kuuspu M. (2018). Effect of Competition and Landscape Characteristics on Mesocarnivore Cohabitation in Badger Setts. J. Zool..

[55] Torretta E., Serafini M., Puopolo F., Schenone L. (2016). Spatial and Temporal Adjustments Allowing the Coexistence among Carnivores in Liguria (N-W Italy). Acta Ethol..

[56] Lesmeister D.B., Nielsen C.K., Schauber E.M., Hellgren E.C. (2015). Spatial and Temporal Structure of a Mesocarnivore Guild in Midwestern North America. Wildl. Monogr..

[57] Major J.T., Sherburne J.A. (1987). Interspecific Relationships of Coyotes, Bobcats, and Red Foxes in Western Maine. J. Wildl. Manag..

[58] Kitchen A.M., Gese E.M., Schauster E.R. (1999). Resource Partitioning between Coyotes and Swift Foxes: Space, Time, and Diet. Can. J. Zool..

[59] Bonte D., Keith S., Fronhofer E.A. (2024). Species Interactions and Eco-Evolutionary Dynamics of Dispersal: The Diversity Dependence of Dispersal. Philos. Trans. R. Soc. Lond. B Biol. Sci..

